# Taming active transposons at Drosophila telomeres: The interconnection between HipHop’s roles in capping and transcriptional silencing

**DOI:** 10.1371/journal.pgen.1009925

**Published:** 2021-11-23

**Authors:** Min Cui, Yaofu Bai, Kaili Li, Yikang S. Rong

**Affiliations:** 1 School of Life Sciences, Sun Yat-sen University, Guangzhou, China; 2 Hengyang College of Medicine, University of South China, Hengyang, China; Geisel School of Medicine at Dartmouth, UNITED STATES

## Abstract

*Drosophila* chromosomes are elongated by retrotransposon attachment, a process poorly understood. Here we characterized a mutation affecting the HipHop telomere-capping protein. In mutant ovaries and the embryos that they produce, telomere retrotransposons are activated and transposon RNP accumulates. Genetic results are consistent with that this *hiphop* mutation weakens the efficacy of HP1-mediated silencing while leaving piRNA-based mechanisms largely intact. Remarkably, mutant females display normal fecundity suggesting that telomere de-silencing is compatible with germline development. Moreover, unlike prior mutants with overactive telomeres, the *hiphop* stock does not over-accumulate transposons for hundreds of generations. This is likely due to the loss of HipHop’s abilities both to silence transcription and to recruit transposons to telomeres in the mutant. Furthermore, embryos produced by mutant mothers experience a checkpoint activation, and a further loss of maternal HipHop leads to end-to-end fusion and embryonic arrest. Telomeric retroelements fulfill an essential function yet maintain a potentially conflicting relationship with their *Drosophila* host. Our study thus showcases a possible intermediate in this arm race in which the host is adapting to over-activated transposons while maintaining genome stability. Our results suggest that the collapse of such a relationship might only occur when the selfish element acquires the ability to target non-telomeric regions of the genome. HipHop is likely part of this machinery restricting the elements to the gene-poor region of telomeres. Lastly, our *hiphop* mutation behaves as a recessive suppressor of PEV that is mediated by centric heterochromatin, suggesting its broader effect on chromatin not limited to telomeres.

## Author summary

Transposons are selfish elements that multiply by inserting extra copies of themselves into the host genome. Active transposons thus threaten the stability of the host genome, while the host responses by transcriptionally silencing the selfish elements or targeting their insertions towards gene-poor regions of the genome. Chromosome ends (telomeres) in the fruit fly *Drosophila* are elongated by active transposition of retrotransposons. Although much is known about how these elements are silenced, little is known about the remarkable accuracy by which they are targeted to telomeres. Prime candidates through which the host mounts such defenses are members of the protein complexes that protect telomeres. Here we characterized a hypomorphic mutation of the HipHop protein, and showed that active telomeric transcription in the mutant germline persists for generations without leading to runaway telomere elongation, that embryos laid by the mutant female suffer rampant end-to-end fusions, and that telomeric targeting of the transposon machinery is defective in the mutant soma. Collectively our data suggest that HipHop is essential for preventing telomere fusions, silencing telomeric transposons, and recruiting transposon machinery to telomeres. Our study thus identifies a factor essential for the host control over active transposons and a paradigm for studying such control mechanisms.

## Introduction

Transposable elements (TEs) are omnipresent in eukaryotic genomes. They are primarily viewed as a threat to the host organism as TE insertions can disrupt gene expression and function, or induce secondary genome instability as a result of illegitimate recombination. The presence of certain TEs is nevertheless beneficial to the host, and one of the best examples concerns telomeres in the model of *Drosophila*, where chromosome ends are populated by telomere specific retrotransposons. These elements serve to elongate chromosome ends thus counteracting sequence loss due to incomplete end replication. In *Drosophila melanogaster*, three classes of non-LTR type retrotransposons make up telomeric DNA: *HeT-A*, *TART* and the *HeT-A*-related *TAHRE*, with *HeT-A* being the most abundant class [1–3]. *HeT-A* has a single open-reading-frame (orf) encoding a Gag-like protein (Orf1p) but lacks the accompanying *orf2*, encoding the reverse transcriptase found in typical non-LTR elements. This suggests that *HeT-A* lacks the ability to transpose on its own. *TART* and *TAHRE* each carry both *orf*s. Whether they control *HeT-A* transposition in addition to their own is not known. Full-length elements are not found elsewhere in the genome suggesting that their transposition is limited to chromosome ends. Remarkably, these elements have the ability to attach to a chromosome end that does not terminate on one of the normal telomeric transposons [4] *and references therein*. How this remarkable target specificity is achieved remains poorly understood. In our earlier study [5], we showed that the Orf1p protein from *HeT-A* is present in the nucleus of somatic cells in S phase, and forms large spherical structures that are attached to one and sometimes multiple chromosome ends. These “*HeT-A* Spheres” consist of a proteinaceous shell made of Orf1p that encapsulates sense transcripts from *HeT-A*. We speculated that *HeT-A* Sphere represents an intermediate in the molecular events leading to *HeT-A* transpositions.

In addition to serving a function of timely elongation of chromosome ends, telomere performs another essential function: to prevent the recognition and illicit repair of telomeres as broken DNA ends. This capping function relies on protein complexes that are specifically enriched on telomeric chromatin: the capping complex. In *Drosophila*, we and others identified two potentially separate complexes essential for the prevention of telomere fusion. First, occupying primarily the double stranded portion of telomeric chromatin is the HipHop-HOAP complex [6], which includes the previously identified HOAP protein [7,8]. HipHop-HOAP may also recruit Heterochromatin Protein 1 (HP1) to telomeres for transcriptional regulation [6,8]. In addition, telomeres end in a 3’ overhang in most systems studied (reviewed in [9]). In *Drosophila*, we proposed a second Moi-Tea-Ver (MTV) complex occupying this overhang based on *in vitro* results [10]. MTV includes the Tea protein [10], and the Moi and Ver proteins previously identified [11,12]. We showed that the Ver subunit of MTV, and quite possibly the other two, is required for *HeT-A* Sphere formation on telomeres [5], and proposed that this single-strand binding complex participates in the recruitment of transposon RNP to *Drosophila* telomeres [10]. This would be similar to the recruitment of telomerase by the CST complexes in other organisms (reviewed in [13,14]). Therefore, *Drosophila* capping machinery likely limits TE attachment only to telomeres, setting up a potential conflict between the two entities. The group of proteins enriched at *Drosophila* telomeres have been collectively called “Terminin” [12], similar to the concept of “Shelterin” proposed for telomerase-maintained systems [15].

Transcriptional regulation is another layer of control placed on telomeric TEs by the *Drosophila* host. Although transposon transcripts [16,17] and proteins [5] were readily detectable in proliferating tissues in the soma, their presence is very low in the germline owning at least partially to the transcriptional regulation mediated by piRNAs (for reviews on transcription regulation at *Drosophila* telomeres, see [18–20]). In piRNA mutant germlines, transcripts from telomeric TEs are overproduced just as the other TEs genome wide. These mutations commonly result in the loss of female fertility. In cases where limited fertility was allowed so that mutant stocks were maintained for many fly generations, an increase of transposon copy numbers at telomeres has been reported (e.g. [21–24]). These results suggest that retrotransposon overexpression might be sufficient for “excess” telomere accumulation of retro-elements. However, there are also cases in which telomere over-elongation was not associated with apparent de-silencing at telomeres (reviewed in [25]). A recent survey of copy number variations in laboratory stocks identified a large range of telomeric array sizes under seemingly wildtype backgrounds [26], confounding the notion that extra copies of telomeric elements might be detrimental to the host. Moreover, converting fitness loss in the laboratory setting to that in nature has always been difficult.

*Drosophila* telomeres display signs of accelerated evolution. First, telomere arrays not only display copy number variations but also a high degree of sequence diversity [26–29]. In the most extreme cases, telomeres can lose all the telomeric elements, either naturally or artificially, and still retain a normal capping function. Moreover, most if not all components of the capping complexes that are specifically enriched at chromosome ends are poorly conserved at the primary sequence level and show signs of fast protein evolution among Drosophilids (e.g. [6,10,12,30]). It is plausible that the fast evolution of telomeric proteins is driven by the highly diverse DNA sequences underlying fly telomeres. However, since the function of the capping complex requires no DNA sequence component, it is difficult to formulate a model in which highly variable DNA sequences drive the evolution of proteins that bind to them. In addition, the difficulty in extracting any fitness consequence in stocks with extra-long telomeres seems to suggest that the host plays a passive role in the evolutionary arm race with the TEs. How the host capping complexes limit telomere TEs propagation is largely unclear since strong loss of their functions invariably impair viability. Recently, Saint-Leandre et al. [24] conducted the first study in which a replacement of HOAP protein of *D*. *melanogaster* with one from its close relative *D*. *yakuba* resulted in transposon de-repression in the germline.

Here we showed that a novel *hiphop* mutation in *D*. *melanogaster* leads to a specific de-repression of telomeric elements in the germline via a mechanism that is largely independent of the piRNA-mediated pathway but possibly dependent on HP1 functions. Remarkably, this de-repression did not result in “extra-long” telomeres, most likely because the mutation simultaneously makes HipHop less effective in recruiting the elements to chromosome ends. Although our study was primarily designed to investigate the mechanisms by which the capping machinery regulates telomeric transposons, our results nevertheless shed new insights into the evolutionary relationship between the host and the TEs. We speculate that the ability of the capping machinery to target all transpositions to chromosome ends represents the most important weapon in the host’s response to the presence of these active transposons.

## Results

### A new *hiphop* mutation causes female sterility

**Fig 1:**
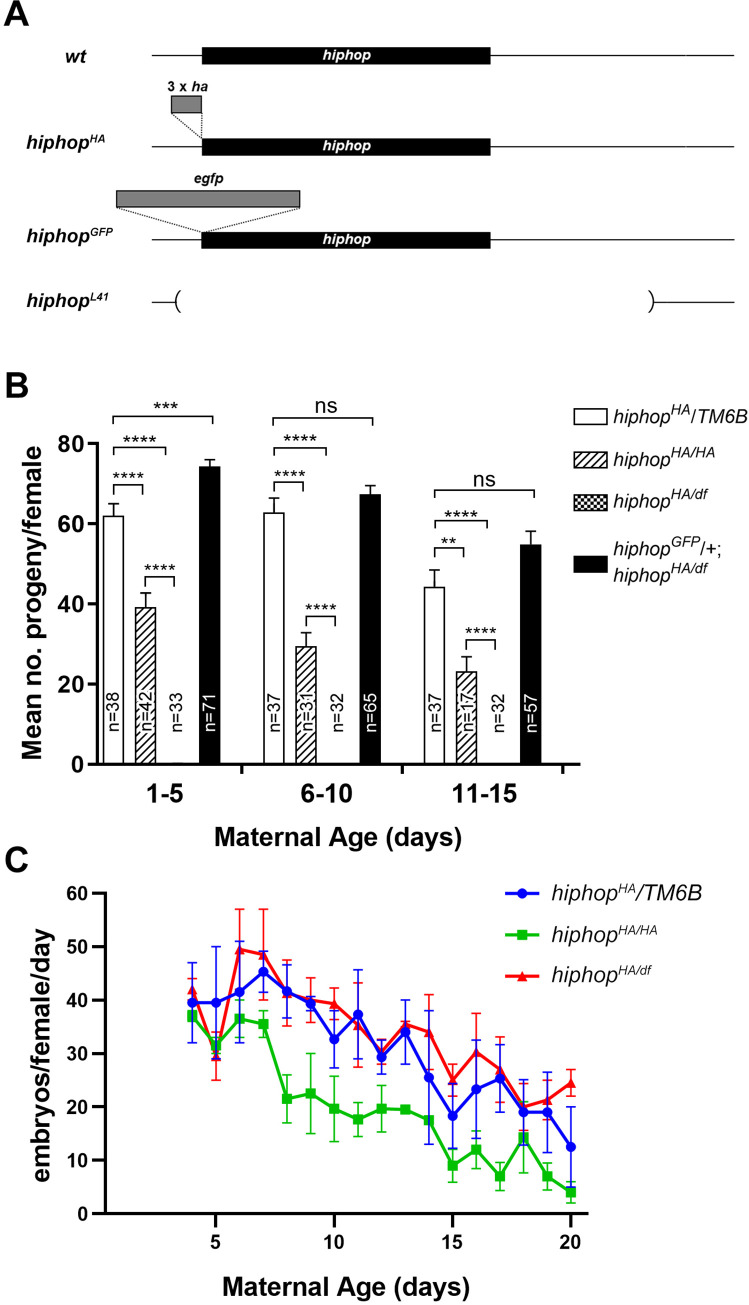
The *hiphop*^*HA*^ mutation affects female fertility. **A.** *hiphop* alleles and constructs. At the top is the wildtype (*wt*) *hiphop* locus with the coding region shown as a black rectangle. The approximate position of the 3xHA tag is indicated for the *hiphop*^*HA*^ allele. The approximate position of the EGFP tag is indicated for the *hiphop* genomic construct used in the rescue experiment. The approximate position of the region deleted in the *hiphop*^*L41*^ allele is indicated by a parenthesis. **B**. The number of progenies produced in relation with maternal ages. The four genotypes tested are shown at the top right. Maternal ages are grouped into three categories and shown as the X axis. The number of females tested (n) is shown for each genotype. For *hiphop*^*HA/df*^ mothers, the progeny counts were zero regardless of maternal age. ns: not significant; **: p<0.01; ***: p<0.001; ****: p<0.0001. **C**. The number of embryos produced in relation with maternal ages. For each genotype (shown at the top right), three separate measurements with ten females each were conducted. *df*: *Df(3L)Cat*, which is a deficiency of the *hiphop* region.

During our studies of the function of HipHop, we observed that putting a 3XHA tag on the N-terminus of Hiphop generated a mutant allele, and named it *hiphop*^*HA*^ (Fig 1A). Previously we showed that *hiphop* deletion mutations are homozygous lethal at an early larval stage [31]. *hiphop*^*HA*^ homozygotes (genotype: *hiphop*^*HA/HA*^), on the other hand, survived at Mendelian ratio (see S1 Table for progeny counts), with no visible phenotypes except that females had reduced fertility (Fig 1B), while male fertility is not affected (n>200). In addition, *hiphop*^*HA*^ hemizygous females are sterile (Fig 1B). These females carry *hiphop*^*HA*^ on one chromosome *3* and a deficiency (*df*) of the *hiphop* region on the homologous chromosome (genotype: *hiphop*^*HA/df*^). Moreover, females trans-heterozygous for *hiphop*^*HA*^ and *hiphop*^*L14*^ (an *hiphop* deletion mutation [31]) were also sterile. The fertility of *hiphop*^*HA*^ homozygotes or hemizygotes was not significantly affected by the maternal age as shown in Fig 1B and 1C. We observed a drop in the number of embryos laid by *hiphop*^*HA/HA*^ mothers. We believe that this might have been caused by second-site mutations in the stock as the hemizygotes have wildtype level of fecundity (Fig 1C). Therefore, *hiphop*^*HA*^ is a recessive mutation that affects female fertility in a dosage dependent manner. To further confirm that the sterility was indeed due to a loss of HipHop function, we introduced a transgene carrying a wildtype copy of *hiphop* and rescued female sterility (Fig 1B).

We used RT-qPCR to investigate whether the *hiphop*^*HA*^ mutation reduces *hiphop* transcript level. As shown in S1A Fig, *hiphop*^*HA*^ results in an approximately 50% reduction of *hiphop* transcript. Since a *hiphop* deletion heterozygote, which does not show phenotypes, is expected to have a similar expression level as that of *hiphop*^*HA/HA*^, we suspect that the HipHop^HA^ protein itself might be defective. Although we don’t know the exact nature of the molecular defect, we note at least one precedent in which an HA tag disrupts the function of the target protein [32].

### *hiphop*^*HA*^ de-represses telomeric retrotransposons in the female germline

The female specific sterility of *hiphop*^*HA*^ mutants suggests defects during oogenesis that might be unrelated to telomere capping. Transposons in *Drosophila*, including those at the telomeres, are silenced in the female germline. We therefore investigated whether telomeric elements are properly silenced in the mutant ovaries.

**Fig 2:**
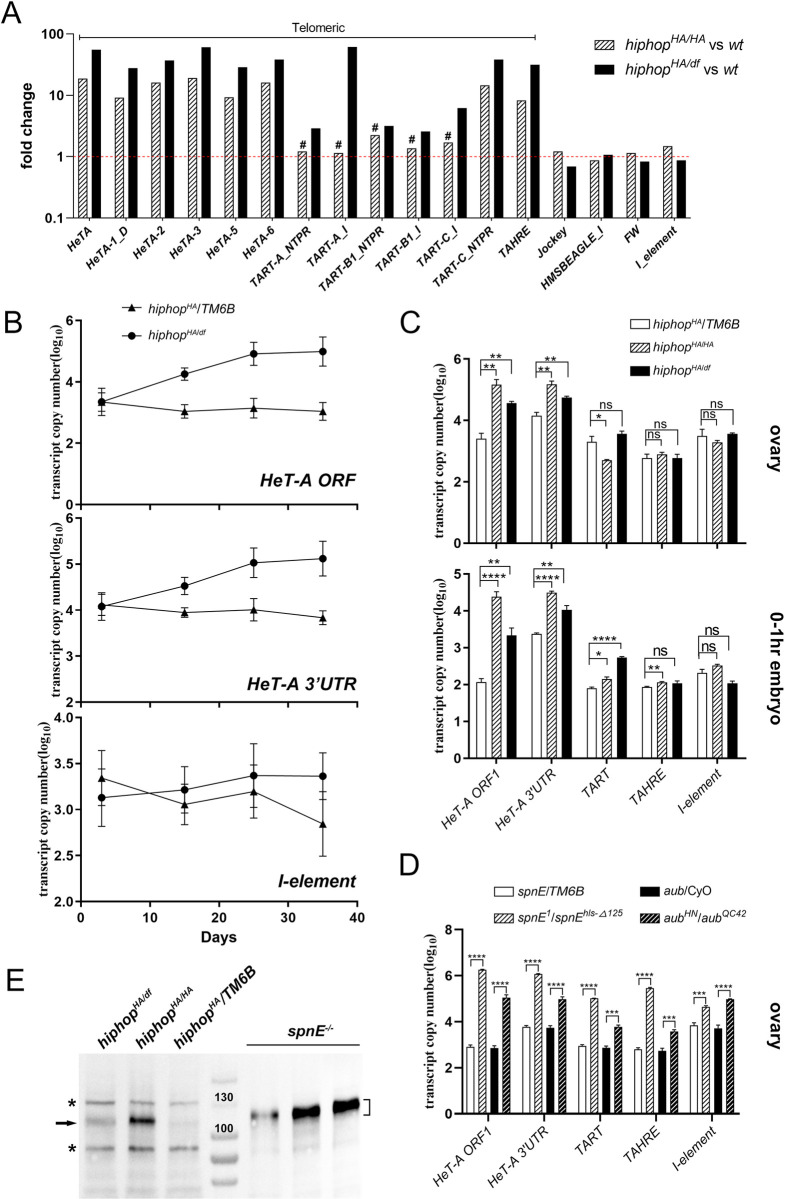
*hiphop*^*HA*^ de-represses telomeric retrotransposons in the female germline. **A**. Fold changes between mutant and wildtype of all telomeric and four non-telomeric TEs are plotted for two genotype pairs. Except for the elements marked with # and under the *hiphop*^*HA*^ background, telomeric TEs are significantly de-repressed in the mutants. Non-telomeric TEs do not show significant differences between the mutants and wildtype. Note that the Y axis is on a log_10_ scale, and it is not drawn to scale. **B**. The effect of maternal age on TE de-repression in *hiphop*^*HA/df*^ mutants. The expression levels of the *orf* region of *HeT-A* (top), the *3’UTR* region of *HeT-A* (middle) and the *I* element (bottom) are plotted according to the ages of the females from the mutant or their heterozygous siblings. **C**. TE levels in *hiphop* mutants and their heterozygous siblings. Transcript levels from three telomeric elements and the non-telomeric *I* element were measured in ovarian (top chart) and 0-1hr embryos (bottom chart) samples. **D**. TE levels from piRNA mutants and their heterozygous siblings. Transcripts were measured for the same elements as those in **C**. **E**. Western blot on ovarian extracts. Membrane was probed with an anti-Orf1p antibody. Genotypes are listed at the top. The three samples from the *spnE*-mutant germline are different dilutions of the same extract (from left to right: 1/20, 1/4, 1/1). The approximate running position of Orf1p is indicated by an arrow at the left and a bracket at the right. Asterisks mark non-specific bands, which also serve as loading controls. The positions for the 100 KD and 130 KD markers are indicated. ns: not significant; *: p<0.05; **: p<0.01; ***: p<0.001; ****: p<0.0001.

We performed whole genome RNA sequencing using ovarian RNA from 15-day old *hiphop*^*HA/+*^ and both *hiphop*^*HA/HA*^ and *hiphop*^*HA/df*^ females. As shown in Fig 2A, RNA seq identified a defect in transposon silencing that is limited to TEs at telomeres: *HeT-A*, *TART*, *TAHRE*. An expression analysis of other TEs in the genome is shown in S2 Table. To facilitate further investigation into the potential mechanisms underlying this de-repression, we employed a qPCR assay in subsequent analyses with primers previously used for the detection of specific TEs [33,34]. For *HeT-A* elements, our qPCR primers anneal to two separate regions. To control for possible age-related effects, we used age-matched samples from the three genotypes of interest (*hiphop*^*HA/+*^; *hiphop*^*HA/HA*^; and *hiphop*^*HA/df*^). As shown in Fig 2B, the mutant germline shows a gradual increase of *HeT-A* transcripts when compared with the heterozygous control, resulting in cumulative ~40-180-fold increase at a maternal age of about 35 days. On the other hands, the *I* element, a non-telomeric non-LTR retrotransposon in the *Drosophila* genome [35], is not de-repressed regardless of the age of the females. Based on these preliminary results, we used RNA samples from 15-day old ovaries in subsequent characterizations. As shown in Fig 2C, although de-repression of *HeT-A* could be demonstrated most consistently, transcription of *TART* or *TAHRE* showed more variable results using the method of qPCR. We suggest that these variations are due to the fact that both *TART* and *TAHRE* are minor elements, and that their sequence variations might be less compatible with our primers. Nevertheless, the non-telomeric *I* element consistently showed a lack of silencing defects in these independent trials. These different responses from telomeric *versus* non-telomeric elements to the *hiphop* mutation are in sharp contrast to the situation in piRNA-mutant germlines. In *aub* and *spnE* mutant ovaries, all tested transposons are de-repressed (Fig 2D). In addition, the telomeric elements are de-silenced to a greater extent in the piRNA mutant than in *hiphop* mutant ovaries (compare Fig 2C with 2D, note the log scale of the y-axis). Therefore, the *hiphop*^*HA*^ mutation leads to a specific de-silencing of telomere retrotransposons in the germline. Interestingly, the level of telomeric transcript is also elevated in the embryos produced by *hiphop* mutant females suggesting that they are maternally deposited (Fig 2C).

The *HeT-A* element encodes a single Orf1p protein. In Western blots using total extracts from ovaries (one shown in Fig 2E), we detected a marked increase of Orf1p level in both of the *hiphop* mutant backgrounds using an anti-Orf1p generated previously [5]. The piRNA-defective ovary displays an even higher increase of Orf1p level (Fig 2E), similar to qPCR results measuring transcript levels. Therefore, at least some of the transcripts generated as a result of de-silencing are capable of being translated.

In contrast to its effect on female fertility (Fig 1B), *hiphop*^*HA*^’s defect in silencing does not display a dosage effect in the qPCR assay (Fig 2C), despite that it seems to be the case in our RNA seq assay (Fig 2A). This difference could be again due to elevated sequence variations of telomeric elements in different genetic backgrounds affecting primarily the qPCR assay.

In summary, the *hiphop*^*HA*^ mutation impairs female fertility, and the silencing of telomere retro-elements. This de-silencing also leads to an elevated production of transposon encoded proteins.

### Transposon de-repression is specific to chromosome ends but independent of global piRNA functions

**Fig 3:**
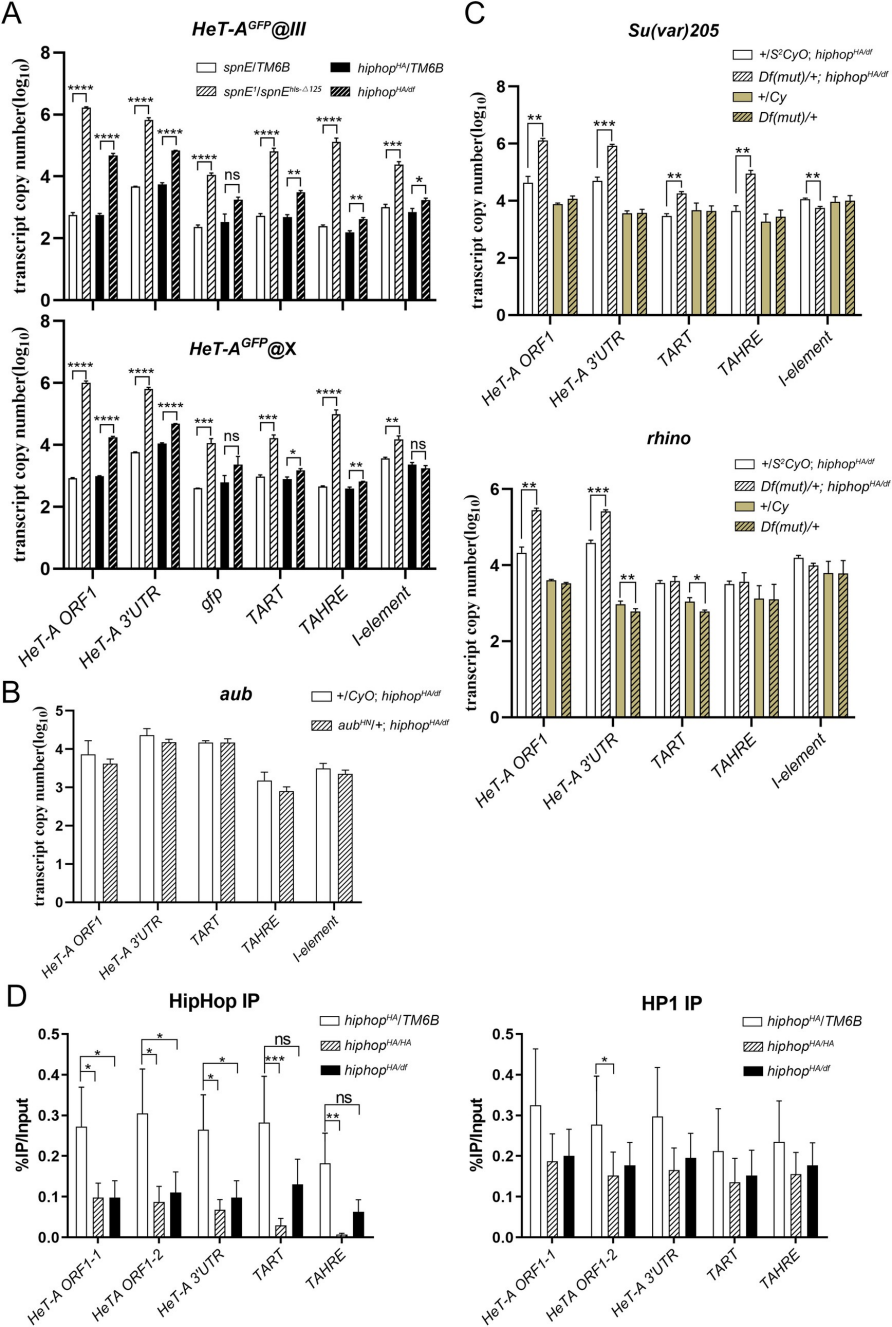
Transposon de-repression in *hiphop*^*HA*^ germline is telomere specific but piRNA independent. **A**. TE levels in *hiphop* and piRNA mutants. Ovaries were taken from adults with a transgenic *HeT-A* element marked with *orf1p-gfp* inserted at non-telomeric positions on chromosome *3* (*HeT-A*^*GFP*^*@III*) or the *X* chromosome (*HeT-A*^*GFP*^*@X*). Transcript levels from four telomeric regions and two non-telomeric regions: *I* elements and the transgenic *HeT-A* (*gfp*) were measured and plotted for two mutants and their heterozygous siblings. Note that the *gfp*-marked elements were de-repressed in *spnE* but not *hiphop*^*HA/df*^ mutant germlines. **B**. Genetic interaction between piRNA and *hiphop* mutants. Transcripts from *hiphop* mutants, and *hiphop* mutants that were also heterozygous for *aub* were measured in ovarian samples. **C**. Genetic interaction between *hiphop* mutants and mutants of HP1-like proteins. Transcripts from *hiphop* mutants, and *hiphop* mutants that were also heterozygous for either *Su(var)205* (top) or *rhino* (bottom) were measured in ovarian samples. For *Su(var)205*, *Df(mut)* = *Df(2L)BSC227*; for *rhino*, *Df(mut)* = *Df(2R)Exel7149*. **D**. Chromatin immunoprecipitation of HipHop and HP1 at telomeric elements. Chromatin occupancy of HipHop and HP1 proteins was assayed by ChIP followed by qPCR on five regions on telomeric elements for two mutants and their heterozygous siblings. ns: not significant; *: p<0.05; **: p<0.01; ***: p<0.001.

The extent of telomere de-silencing in *hiphop*^*HA*^ mutants is remarkable if one considers that it happens under the background of an intact piRNA pathway. To help identify potential mechanisms, we first addressed the question of whether HipHop-mediated silencing is specific to the retrotransposon *per se*, regardless of where the elements are positioned in the genome. This is relevant because we and others have shown that transcripts generated from transgenes under the regulatory control of *HeT-A* were greatly elevated in a piRNA defective germline, even when the transgenes are not inserted at a telomeric position [36,37]. If such a transgene were similarly de-repressed under the *hiphop*^*HA*^ mutant background as its endogenous counterparts at the telomeres, we would conclude that HipHop acts on *HeT-A* specifically. As shown in the qPCR results in Fig 3A, this does not appear to be the case. The endogenous *HeT-A* elements are de-repressed in either a *hiphop* or a piRNA defective background (*spnE*^*-*^), while a *gfp*-marked *HeT-A* transgene is only de-silenced under the latter since the slight elevation of *HeT-A*^*GFP*^ expression under the *hiphop*^*HA/df*^ background did not reach statistical significance (Fig 3A). This is shown for two independent *HeT-A*^*GFP*^ insertions on chromosomes *X* and *3*. Therefore, we suggest that HipHop exerts its silencing effect *in cis*.

The above results provide the first line of evidence suggesting that HipHop-mediated telomeric silencing in the germline is independent of the global piRNA pathway. We gathered additional evidence in support of this proposition from a genetic interaction study in which we compared transcript levels of telomeric elements under a *hiphop*^*HA/df*^ background with those under the same *hiphop*^*HA/df*^ background that was also heterozygous for an *aub* mutation. As shown in Fig 3B, this reduction of *aub* dosage has no effect on telomeric silencing brought about by the *hiphop* mutation. In contrast, when we conducted a similar genetic study with a reduction of either HP1 or its germline specific paralog Rhino [38], we observed a further enhancement of *HeT-A* de-silencing (Fig 3C).

The result from using the *rhino* mutant is not consistent with the view that HipHop-mediated silencing at telomeres is completely independent of the piRNA function as Rhino has been well established as an important piRNA protein essential for piRNA cluster specification [38–40]. Instead, we suggest that the *hiphop*-mutant germline may be partly defective in the same process. Nevertheless, our results are consistent with that HP1-like proteins are important player(s) in HipHop-medicated silencing at telomeres. Since HipHop is an HP1 interacting protein [6], we propose that the *hiphop*^*HA*^ mutation might have weakened the binding of HP1 to telomeric chromatin, leading to de-repression. This de-repression would be specific to telomeres as HP1 binding to other regions of the genome is not expected to be affected by the *hiphop* mutation. To test this hypothesis, we measure HP1 occupancy at telomeres using chromatin immunoprecipitation (ChIP). As shown in Fig 3D, while the occupancy of the HipHop^HA^ protein at *HeT-A* elements is reduced compared with that of the wildtype protein, HP1 occupancy at the same elements is generally reduced but the differences fail to achieve statistical significance. Therefore, although our results support a genetic role of HP1 in the process of telomeric de-silencing, molecular evidence remains missing.

### Telomere uncapping leads to maternal lethality of *hiphop*-mutant embryos

**Fig 4:**
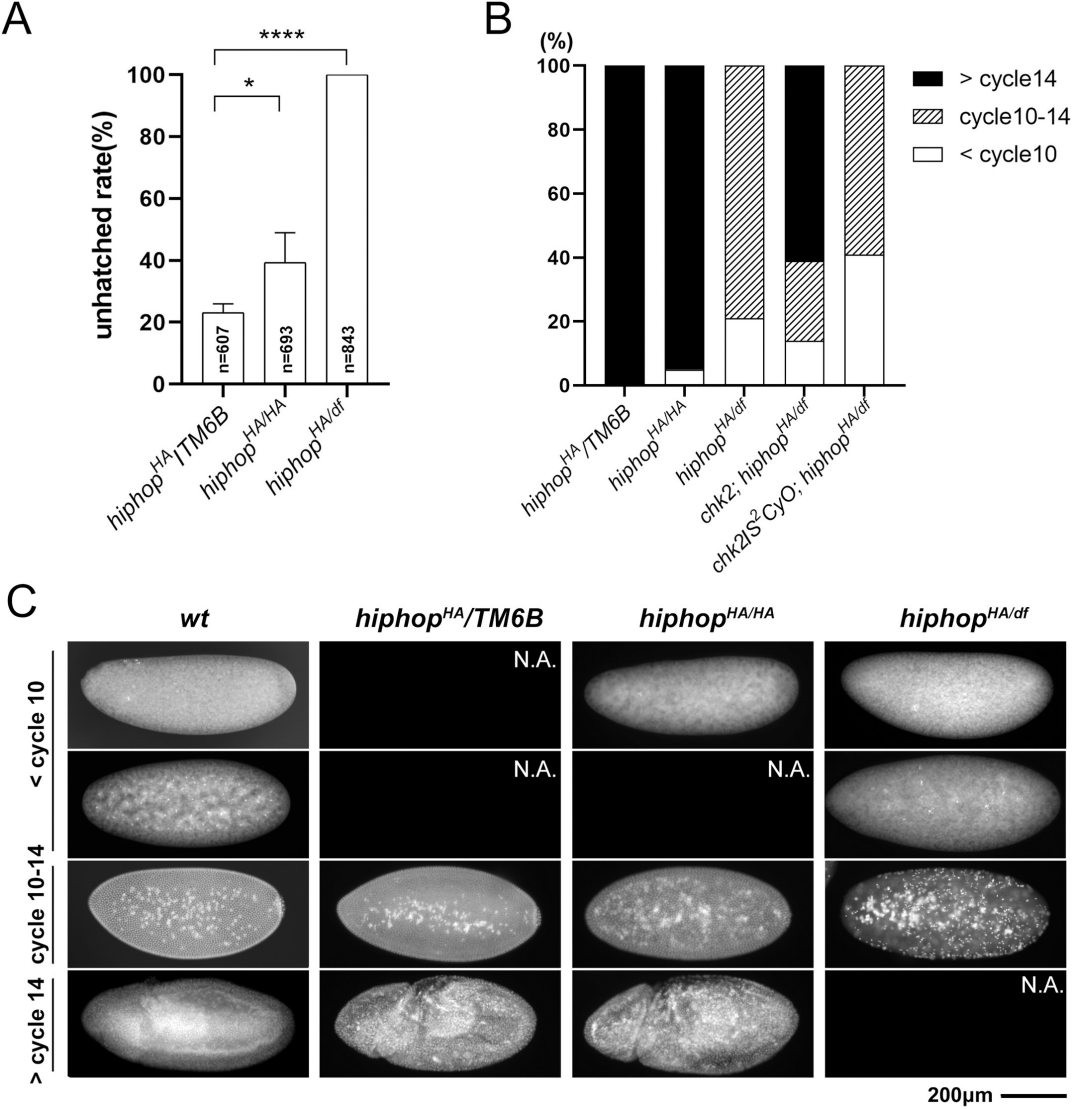
*hiphop*^*HA*^ causes maternal effect embryonic lethality. **A**. Hatching rates of embryos from *hiphop* mutant females and their heterozygous siblings. For each genotype, at least 30 females were tested. **B**. Quantifications of developmental arrest of *hiphop*-mutant embryos. Fixed embryos were scored for their approximate stage of development based on DAPI staining. For each genotype, at least 210 embryos were examined. **C**. Representative images of DAPI-stained embryos at various early stages. Developmental stages are indicated to the left, maternal genotypes at the top with *w*^*1118*^ used as *wt*. N.A.: not available, embryos at the indicated stage were not found.

Although ovaries from *hiphop*^*HA/df*^ animals have high levels of transposon RNP, they do not show discernable defects in morphology. In addition, these females when mated with wildtype males show normal fecundity (Fig 1C), yet very few of the embryos hatched (Fig 4A). We therefore suspected that the *hiphop*^*HA*^ mutation causes maternal effect lethality so that those embryos died due to defective maternal contribution of the HipHop function. We named these embryos as *hiphop*-mutant embryos for simplicity reason, but note that they were genetically heterozygous for a *hiphop* mutation, since they had wildtype fathers.

**Fig 5:**
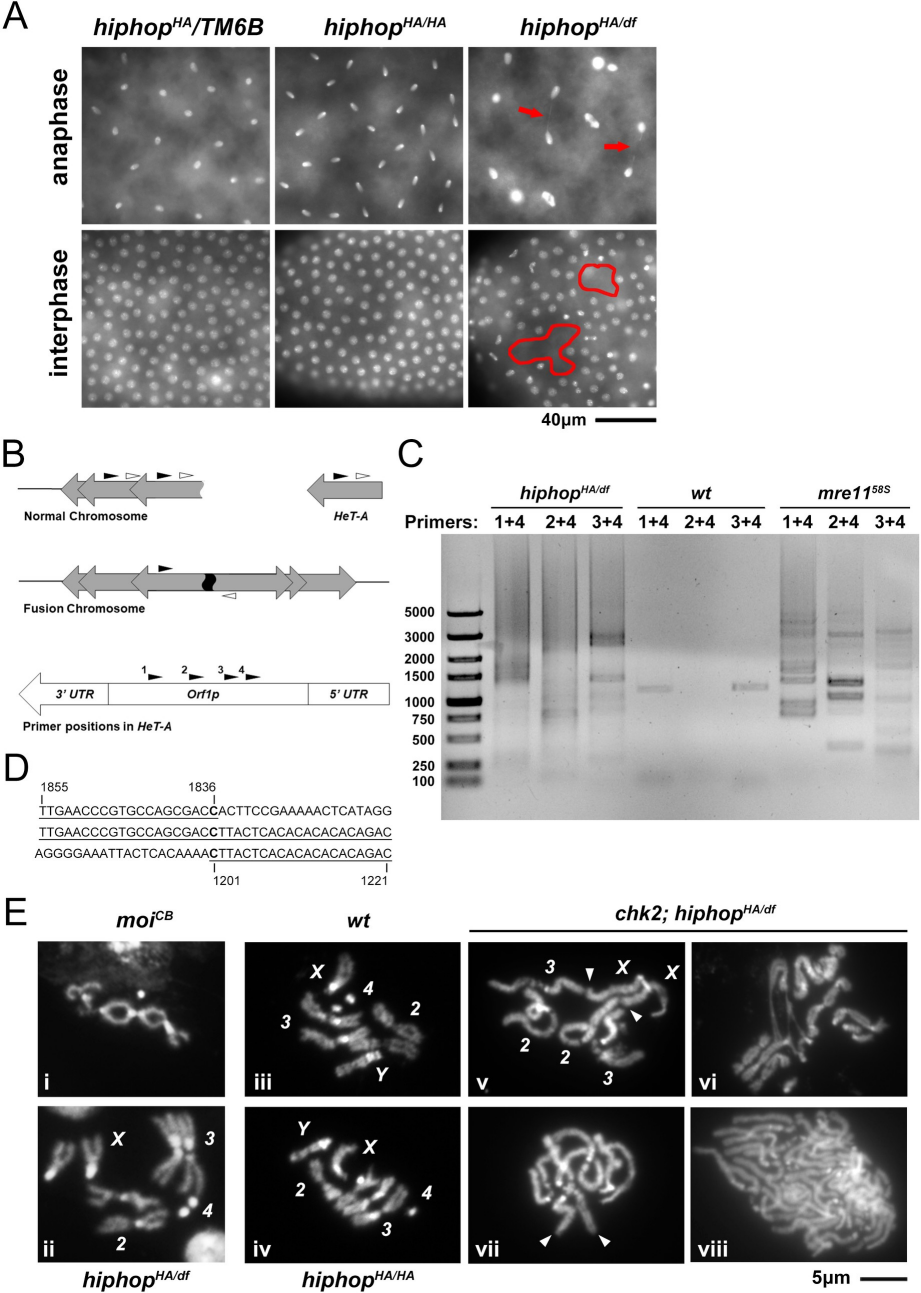
Telomere uncapping in *hiphop*-mutant embryos. **A**. Nuclear defects in *hiphop*-mutant embryos. In *hiphop*^*HA/df*^ images, arrows indicate chromosome bridges (top) and the areas with missing nuclei are demarcated by red lines (bottom). **B**. The scheme for isolating telomere fusion DNA junctions. Block arrows indicate telomeric elements. Half arrows indicate PCR primers and their direction. The approximate positions of the four primers are indicated along a *HeT-A* element at the bottom. **C**. A DNA gel showing PCR products from a fusion PCR reaction. Maternal genotypes and primer combinations are listed at the top. For primer positions, see **B**. Markers with sizes indicated are shown to the left. **D**. The DNA sequence of a fusion junction from *hiphop*-mutants. The Genbank entry S56442.1 was used for sequence alignment, and the numbers are nucleotide numbers in S56442.1. The DNA sequence of the fusion junction (middle line) aligns with two different regions from S56442.1 (top and bottom lines), but at opposite directions. This identifies the nucleotide “C” (in bold) at the fusion junction. **E**. Chromosome configuration from larval neuroblasts (i, ii) and early embryonic nuclei (iii-viii). (i) A nucleus homozygous for the *moi*^*CB*^ mutation, showing telomere fusions. (ii) A nucleus from a *hiphop*^*HA/df*^ larva with all chromosome pairs labelled. The maternal genotypes for the nuclei are (iii): *w*^*1118*^; (iv): *hiphop*^*HA/HA*^; (v)-(viii): *chk2; hiphop*^*HA/df*^. Chromosomes are indicated in (iii)-(v). In (v), fusions between an *X* and *2*, and an *X* and *3* are indicated by arrowheads. In (vi), majority of the chromosomes participates in at least one telomere fusion. In (vii), a long chromosome chain was formed by end fusions with the start and the end of the chain indicated by arrowheads. The nuclei in (viii) are polyploid possibly due to failure of chromosome segregation.

Consistent with our hypothesis, when we collected *hiphop*^*HA/df*^-mutant embryos between 0 and 2hr after they were laid and aged them for an additional two hours, we observed that essentially all of them arrested before cycle 14 of the syncytial divisions despite showing variable extents of development based on DAPI staining (Fig 4B and 4C). Interestingly, a significant but much smaller portion of the embryos (5%) laid by *hiphop*^*HA/HA*^ mothers also arrested at a similar stage, consistent with the homozygotes only having reduced fertility. Upon closer inspection of the arrested embryos, we observed defective nuclear morphologies that include nuclei of abnormal sizes, asynchrony in nuclear divisions, nuclear fallouts leading to large areas without nuclei, and chromosome bridging (Figs 4C and 5A). Therefore, the cause for the early arrest of *hiphop*-mutant embryos is at least partly due to defects in mitosis including chromosome mis-segregation.

Strong loss-of-function mutations of *Drosophila* telomere capping proteins, including HipHop, lead to end-to-end fusions in proliferating cells (e.g. [6,7,10]). Proliferating cells of *hiphop*^*HA/df*^ animals do not display telomere fusions (Fig 5E), suggesting that the *hiphop*^*HA*^ mutation does not impair telomere capping in the post-embryonic soma. Consistently, HipHop^HA^ and its interacting HOAP protein localize to telomeres normally in immunostaining experiments (S1 Fig). At the organismal level, this lack of capping defect is consistent with the mutants surviving into adults with normal appearance. We have shown previously that hypomorphic mutations in capping regulators produce viable and normal looking adults. Yet, they inevitably lead to maternal effect lethality similar to what we observed for *hiphop*^*HA/df*^. A common defect in embryos from those capping hypomorphs is the presence of rampant end-to-end fusion so that early nuclear cycles are greatly disrupted [41,42]. The presence of chromosome bridges in *hiphop*-mutant embryos led us to investigate whether defective telomere capping during early proliferation is the leading cause for lethality.

**Fig 6:**
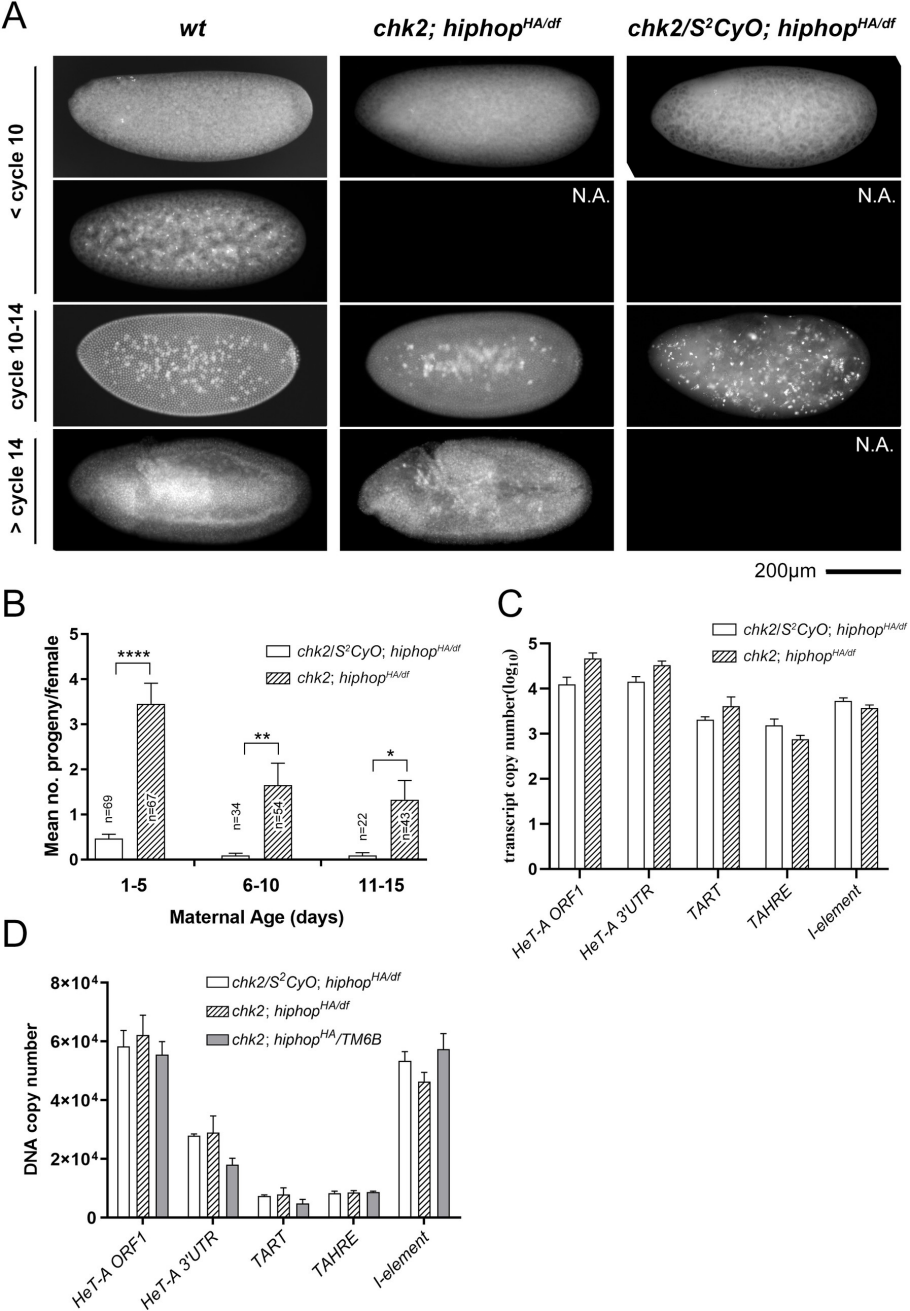
Chk2 activation contributes to developmental arrest of *hiphop*-mutant embryos. **A**. DAPI- stained embryos showing various degrees of developmental progression. Developmental stages are indicated to the left, maternal genotypes at the top. For each genotype, at least 160 embryos were examined. N.A.: not available, embryos at the indicated stage were not found. **B**. A *chk2* mutation partly rescues lethality of *hiphop*^*HA/df*^-mutant embryos. The number of progenies is plotted according to maternal age and genotype. For each genotype, at least 33 of female parents were examined. **C**. The *chk2* mutation does not enhance telomeric transcription in the germline. Transcript levels from four telomeric regions and the *I* element were measured for *chk2 hiphop* double mutants and their heterozygous siblings. **D**. Transposon copy numbers in *hiphop* and *chk2* mutant embryos. DNA qPCR was used to measure transposon copy numbers in embryos laid by females, whose genotypes are indicated at the top.

We first applied a PCR-based method to isolate DNA fragments spanning the fusion junction. Telomeric retrotransposons are arranged in directed repeats so that a pair of primers both aiming towards the chromosome end would not be productive in PCR amplification using wildtype genomic DNA as templates. However, in the event of telomere fusions, the same primer pair could be used productively in genomic PCR (Fig 5B). This method was successfully used by us to molecularly characterize end-to-end fusions in *Drosophila* [41,42]. Using genomic DNA from embryos laid by *mre11*^*58S*^ mutant mothers, we repeated the fusion PCR and recovered abundant products. As shown in Fig 5C, PCR reactions templated over genomic DNA from *hiphop*-mutant or *mre11*^*58S*^ mutant embryos were much more robust than ones over the control template (from embryos laid by *mre11*^*58S*^ heterozygous mothers). When we cloned and sequenced PCR products from all three templates, we recovered 9 out of 12 clones from *hiphop*^*HA/df*^-mutant sample, 13 out of 16 clones from *mre11*^*58S*^ mutant sample, and 0 out of 8 clones from wildtype template, that have signatures of telomere fusion (Fig 6D).

To further confirm the presence of telomere fusions in *hiphop*-mutant embryos, we employed a second method based on cytological analyses of mitotic chromosomes, which was derived from a similar method for visualizing mitotic chromosomes from larval neuroblasts [41]. We failed to produce satisfactory results when applying it to *hiphop*^*HA/df*^-mutant embryos. We reasoned that this was due to the low number of mitotic nuclei caused by the early arrest of those embryos (Fig 4B). We therefore took advantage of the *chk2*-mutant background in which we were able to improve cell cycle progression in the same embryos (see the next section for details). In these embryos laid by females that were *chk2; hiphop*^*HA/df*^, chromosome configurations indicative of end-to-end fusion were clearly identified (Fig 5E). We observed fusion in 95% of the nuclei (n = 94) from the mutant embryos, but none from *hiphop*^*HA/HA*^ (n = 95) or wildtype (n>100) embryos. These results suggest that telomere fusions are ongoing in *hiphop*-mutant embryos, which likely accounts for the lethality.

### Checkpoint activation contributes to lethality of *hiphop*-mutant embryos

The Chk2 protein participates in the response to transposon de-repression in the germline in that a *chk2* mutation often alleviates germline defects associated with transposon de-silencing (e.g. [43,44]). In the *hiphop*^*HA/df*^ germline, loss of Chk2 does not alleviate telomeric de-repression (Fig 6C). We then set out to determine whether Chk2 contributes to maternal lethality caused by the *hiphop*^*HA*^ mutation. As shown in Fig 4B, *hiphop*^*HA/df*^ mutant mothers that were also mutant for *chk2* laid embryos that developed further than those laid by *chk2* heterozygous sisters. Remarkably, some of these Chk2-deficeint embryos survived to adulthood at all three maternal age ranges tested (Fig 6B). These results are consistent with Chk2 being activated in HipHop-deficient embryos and its activation contributing to their developmental arrest. The enhanced developmental progression observed in these doubly mutant embryos is more likely due to suppression of checkpoint activation than to inhibition of telomere fusion, as we showed previously that Chk2 inactivation does not inhibit end fusion [45].

It is known that Chk2 in early embryos can be activated by the presence of DNA damaging or damage-mimicking agents including single-stranded nucleic acids [46]. Our qPCR results shown in Fig 2C suggest that abundant telomeric transcripts are present in early mutant embryos, suggesting that these transcripts activate Chk2. We also considered the possibility that transposition of telomeric elements in these embryos cause secondary genome instability, also activating Chk2. However, this is not supported by the data as we did not observe an increase in DNA copy number of telomeric elements in the mutant embryos (Fig 6D), which would be indicative of active transposition.

### Overactivation of telomeric elements in the absence of run-away telomere elongation

**Fig 7:**
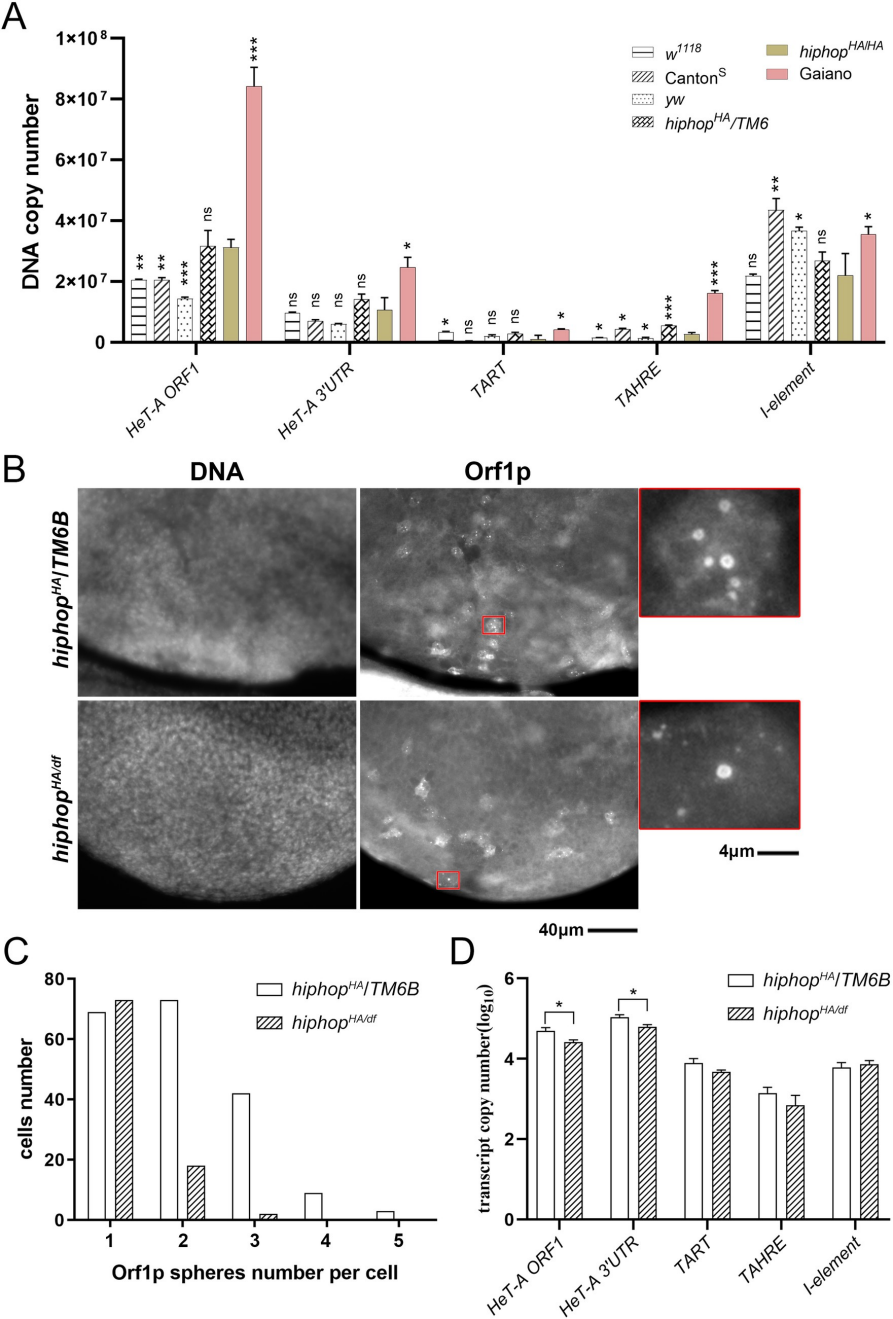
*hiphop*^*HA*^ is incompatible with telomere over-elongation. **A**. DNA copy numbers of four telomeric and one non-telomeric transposons. Samples were from three “wildtype”, the *hiphop*^*HA/+*^, the evolved *hiphop*^*HA/HA*^, and the *Gaiano* stocks. Statistic calls are against the evolved *hiphop*^*HA/HA*^ stock. ns: not significant; *: p<0.05; **: p<0.01; ***: p<0.001. **B**. Orf1p immunostaining. Larval brains were stained with anti-Orf1p and DAPI to identify nuclei with bright Orf1p foci. A close-up view of one nucleus for each genotype is shown. The enlarged area is marker with a red rectangle. **C**. Quantification of Orf1p foci. **D**. TE transcript levels in larvae. Transcripts from four telomeric and the *I* elements were measured for *hiphop* mutants and their heterozygous siblings.

The genetic control for the array length of telomeric transposons remains poorly understood in *Drosophila*. Semi-defined genetic backgrounds have been identified that lead to an accumulation of telomeric elements and discernable elongation of the telomeric arrays cytologically over tens of fly generations. Our *hiphop*^*HA*^ mutation passes two of the preliminary requirements for acquiring an elongated telomeric array: telomeric elements are de-repressed in the germline and the mutant homozygotes are fertile. The original *hiphop*^*HA*^ stock has been kept as a homozygous stock since June of 2010, about 260 fly generations (14 days as an average fly generation) before it was subject to a qPCR-based assay for measuring DNA copy number of the telomeric elements. This is longer than the 50 or so generations used in prior studies for measuring telomere elongation. As shown in Fig 7A, copies of telomeric elements in the “evolved” *hiphop*^*HA*^ stock are not excessively higher than a variety of control stocks. Unfortunately, DNA from the original (“unevolved”) *hiphop*^*HA*^ stock is not available for comparison. As a positive control for DNA copy number measurements, we used the Gaiano stock known to have excessively long telomeric arrays [21].

If one considers the possibility that HipHop serves not only to prevent end fusion but also to control end elongation, the above results might not be surprising. We previously showed that in proliferating cells of a third instar larva, a possible intermediate of *HeT-A* transposition, *HeT-A* Sphere, attaches to telomeres and that this process is defective in mutants of the MTV capping complex [5,10]. Whether HipHop, an essential component of the telomere capping machinery, plays a similar role in the recruitment of transposon RNPs was not examined in the early studies owning to the very early lethality of the null mutant. We therefore investigated whether the *hiphop*^*HA*^ mutation causes a defect in *HeT-A* Sphere formation. In immunostaining results shown in Fig 7B and quantified in 7C, our anti-Orf1p antibody identifies nuclei with Orf1p foci. By counting the number of Orf1p foci in over 100 nuclei per genotype, we identified a significant reduction of Orf1p foci per cell in *hiphop*^*HA/df*^ mutant animals (p<0.0001 from a Mann-Whitney test), suggesting that the *hiphop*^*HA*^ mutation has lost some of HipHop’s ability in mediating *HeT-A* Sphere formation and possibly *HeT-A* transposition to telomeres. Interestingly, *HeT-A* transcripts are moderately reduced in the mutant soma (Fig 7D), which we consider has two possible causes. First, the *hiphop* mutation confers a more efficient silencing of *HeT-A*. Alternatively, the reduction of *HeT-A* Sphere formation renders the transcript less protected. We favor the latter hypothesis since we showed that *HeT-A* RNA resides in the interior of a *HeT-A* Sphere, encapsulated by an outer shell of the Orf1p protein [5]. Lastly, the differences in piRNA and other small RNA-mediated activities between different tissues or different developmental stages might have led to the seemingly opposite effects of *hiphop*^*HA*^ on telomere transcript levels in the female germline *versus* the soma (compare Figs 2C and 7D).

Therefore, we believe that even with an elevated expression of telomeric retrotransposons in *hiphop*-mutant germlines, the mechanism that recruit transposons to chromosome ends is also defective, consistent with *hiphop*^*HA*^ flies failing to acquire elongated telomeric arrays. However, a stronger proof that *hiphop*^*HA*^ limits transposon accumulation would have to come from Orf1p staining in cells where heritable transposition events naturally occur, a process currently not well understood.

### The *hiphop*^*HA*^ mutation is a recessive suppressor of PEV

Although HipHop has been identified as specifically enriched at all telomeres, its localization elsewhere on chromosomes has not been ruled out. In addition, there are prior cases in which changes of telomere structure exert a global effect on transcription (e.g. [47]). We fortuitously uncovered an effect of *hiphop*^*HA*^ on the *white* gene in the *w*^*m4h*^ allele, which experiences variegated expression, Position Effect Variegation (PEV), due to its proximity to centric heterochromatin brought about by an inversion of the *X* chromosome [48].

**Fig 8:**
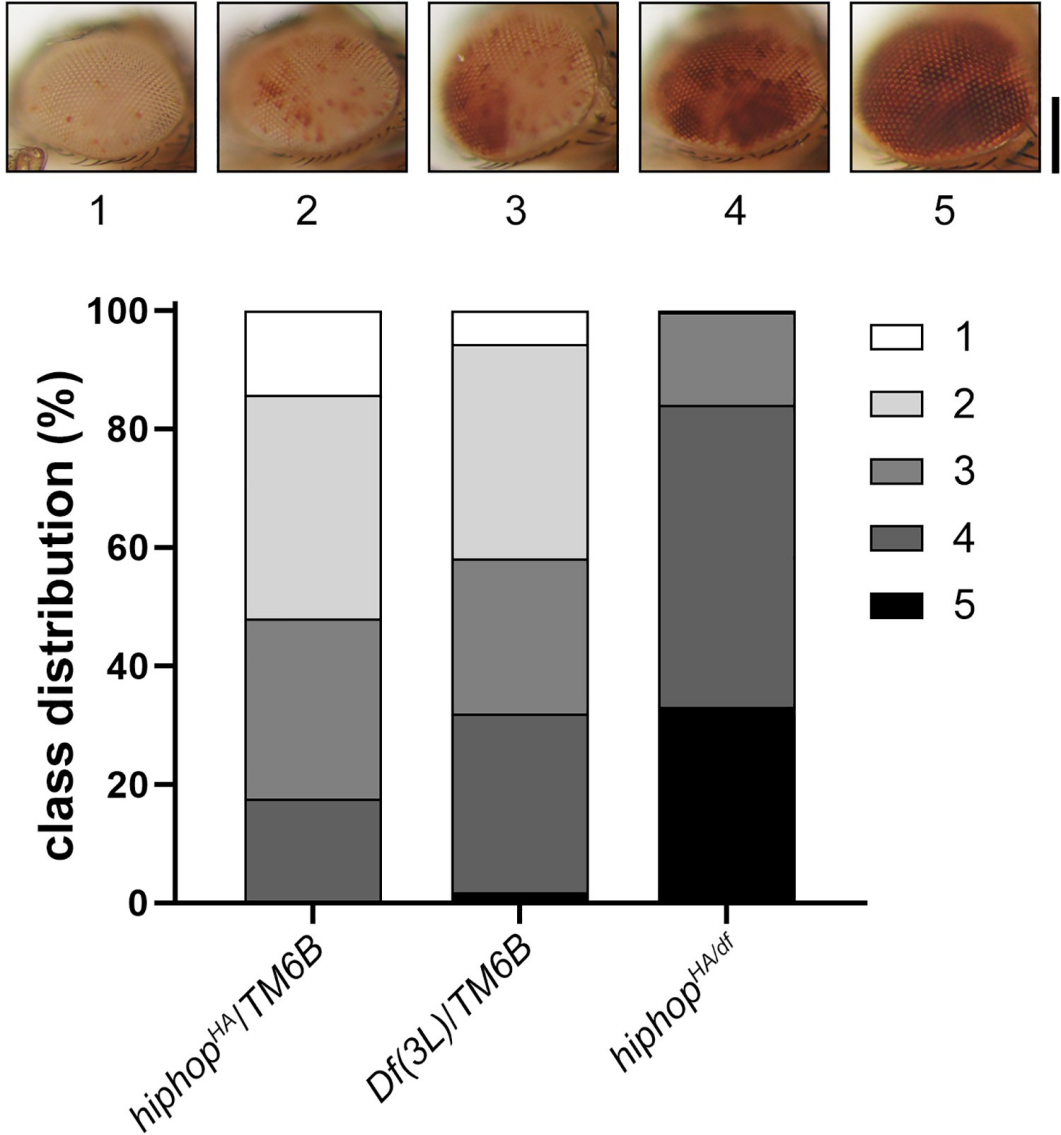
*hiphop*^*HA*^ is a recessive suppressor of PEV. At the top are images of eyes from females with the genotype of *In(1)w*^*m4h*^*/w*^*1118*^ showing various degrees of eye pigmentation. The degree is given a number of 1 to 5 according to the extent of pigmentation. At the bottom is the quantification of eye pigmentation from *hiphop* mutant females and their two kinds of heterozygous sisters. Scale bar indicates 0.2mm.

We classified the degree of *white* variegation into five classes according to the extent of red pigmentation in the eyes, with class I being the most silenced (least pigmentation) and class V being the least (Fig 8). Based on this classification, we discovered that the *hiphop*^*HA/df*^ allelic combination results in the strongest suppression of *white* silencing (most pigmentation) than either of the two *hiphop* alleles in a heterozygous state (Fig 8). Therefore, *hiphop*^*HA*^ suppresses *w*^*m4h*^ PEV as a recessive mutation. This result suggests either that the change of telomeric transcription brought about by the *hiphop*^*HA*^ mutation leads to a global change of chromatin structure *in trans*, particular in heterochromatic regions, or that HipHop is present in centric heterochromatin so that *hiphop*^*HA*^ affects *w*^*m4h*^ transcription *in cis*. Interestingly, a mutation of the HOAP capping protein dominantly suppresses PEV [49].

## Discussion

Telomere in *Drosophila* serves as an excellent model for the study of evolutionary forces that balance the fitness cost of having active TEs with the essential function that these elements fulfill for the host organism. At the interface of this interaction lies the capping complex, the host machinery in direct contact with the TEs, whose subunits are prime candidates through which the host exerts its control. However, it has been difficult to assess the role of capping proteins in transposon regulation, particularly in the germline, as a strong loss of the capping function results in lethality due to end-to-end fusion in somatic cells. An ideal situation for such a study is the availability of separation-of-function mutations that support one but not the other function of the complex. Whether our *hiphop*^*HA*^ mutation represents a true separation-of-function mutation is debatable (see later discussion). It nevertheless supports viability and fertility allowing us to study the effects of transposon hyperactivation on germline and embryonic development, and to infer host mechanisms that mitigates the effect of having active transposons in the genome.

### The increasing complexity of transcriptional regulation of telomeric transposons

Similar to TEs in other parts of the *Drosophila* genome, telomeric TEs are under the piRNA-mediated surveillance in the germline in that TE transcripts serve as precursors for the biogenesis of small RNAs targeting the elements themselves [18–20]. These small RNAs and their associated protein factors control the level of transposon RNAs via two major mechanisms: by directing RNA degradation in the cytoplasm or by maintaining a specialized chromatin structure *in cis* for transcriptional silencing. This second mode of regulation involves the HP1 proteins at telomeres. Given the fact that HipHop is predominantly a telomeric protein, it is likely that the defect(s) leading to transposon de-repression in *hiphop*^*HA*^ germline is *in cis* such as an altered chromatin state at the telomeres. Our genetic results support such a proposition in that a reduction of HP1 dosage further aggravates the silencing defects.

Our results however beg the question of what role the piRNA surveillance plays in transposon silencing under our mutant background. The fact that non-telomeric elements are not de-repressed in *hiphop* mutant ovaries implies that the piRNA mechanisms are fully functional. How could the excess telomeric transcripts escape the surveillance of a functional piRNA machinery? It is possible that the extent of telomeric overexpression exceeds the capacity of piRNA-mediated transcript degradation. We consider this unlikely on the reasoning that telomeric de-repression under a piRNA-mutant background is much greater than that in the *hiphop* mutant germline. Therefore, the capacity of piRNA mediated RNA degradation is not likely to have reached its capacity in our mutant. Alternatively, piRNA production might be defective in our mutant as suggested by our genetic interaction study with the *rhino* mutation. Since this proposed defect is unlikely due to the lack of telomeric transcription *per se*, we suggest that the aberrant telomeric transcripts might not be conducive to piRNA production, or that these aberrant transcripts are themselves resistant to piRNA mediated degradation. We don’t favor this hypothesis, however, since some of the *HeT-A* transcripts are functional in producing the Orf1p protein.

### The effects of excessive transposon RNPs on organismal development

A common organismal phenotype associated with transposon de-repression is the loss of female fertility as demonstrated in many studies of piRNA and related pathways. As transposon RNPs are deposited into the egg, lethality of the resulting embryos has also been reported [33,50]. Since de-repression happens genome-wide and to different classes of elements in these earlier studies, it has been difficult to determine whether any particular class of elements exerts a disproportionally large effect on organismal fitness, or whether the mere presence of excessive amounts of RNA and protein molecules is sufficient to impose the detrimental effect.

We showed that *hiphop*-mutant ovaries specifically accumulate a large amount of telomeric transcripts and the Orf1p protein from *HeT-A*. Therefore, the specific effects of highly active telomeres on development could be studied. Despite telomere hyperactivation in the germline, mutant females laid normal number of fertilized eggs. These results are consistent with ones reported earlier suggesting that telomere specific de-repression does not have a catastrophic effect on germline development [24,51].

Contrary to the lack of effect on female fecundity, the presence of excess telomeric RNPs is associated with defects in embryonic development, and those defects can be rescued partially with a *chk2* mutation suggesting that they were due to checkpoint activation. However, checkpoint activation by excessive transposon RNPs does not necessarily result in embryonic lethality since over 80% of the embryos laid by *hiphop*^*HA*^ homozygous mothers hatch (normalized over the hatching rate from the heterozygotes) even though they too have inherited abundance of transposon RNPs. Therefore, telomere specific de-repression does not necessarily have a catastrophic effect on embryonic development either, suggesting that telomeric over-activation is well tolerated and more sensitive methods are needed to uncover its effects on organismal fitness.

### *hiphop*^*HA*^ is a hypomorphic mutation in telomere capping

Contrary to *hiphop*^*HA*^ homozygous females that have good fertility, majority of embryos laid by *hiphop*^*HA*^ hemizygous mothers arrest very early during development with defects in chromosome segregation. We identified telomere fusion as likely the major cause for this embryonic lethality, which was based on two independent assays of “fusion PCR” and “mitotic chromosome squash”. This contrasts with the lack of capping defects in somatic tissues of the same mutant, which suggests that the HipHop^HA^ protein retains sufficient capping function in post-embryonic somatic cells but not during the rapid embryonic cycles. We showed earlier that hypomorphic mutations in the Mre11-Rad50-Nbs complex or the ATM checkpoint kinase supports normal somatic development but causes severe uncapping in early embryos [41,42]. These results are consistent with the propositions that the syncytial cycles place an exquisite requirement on telomere capping, and that *hiphop*^*HA*^ is a hypomorphic mutation for telomere capping.

We consider it unlikely that telomere uncapping in *hiphop*-embryos is secondary to the primary defect of having hyperactive telomeres. Based on limited cytological evidence, it has been previously claimed that a high level of telomeric transcripts is accompanied by telomere fusions in embryos functionally defective for piRNA [50], or for the degradation of telomeric transcripts [33]. It is difficult to envision how an overabundance of telomeric RNA induces uncapping since the source of these RNAs is primarily maternal. If there were wide-spread telomere uncapping in those transcriptionally inert embryos, it might be more likely caused by the reduction of maternal factors essential for the loading of capping complexes.

### Are the capping and silencing functions of the capping complex separable?

The capping complex could be intimately involved in transcriptional regulation by maintaining a chromatin structure at telomeres, and a common chromatin feature may be essential for both transcription regulation and end capping. In support of this model are the findings that mutations in HP1 impair both processes [52], and that the HipHop-HOAP complex at telomeres contains HP1 [6]. However, the two chromatin domains, one for end protection and the other for transcriptional silencing are not identical. First of all, Biessmann et al. [53] showed that different telomeric domains support different transcriptional activities from the same transgene. Moreover, Wei et al. [26] reported that the size of the telomeric array can vary greatly, up to a 288-fold range in laboratory stocks. Therefore, the machinery essential for transcription regulation likely has a wider and more variable distribution on telomeres than the capping complex, which is limited to the more immediate vicinity of the actual chromosome ends [6]. For example, HP1 and other transcriptional regulators might be initially recruited to chromosome ends by their interactions with the capping complex, and subsequently spread to neighboring regions for transcriptional silencing. In the case of a partial loss of the capping proteins, fewer silencing proteins might be recruited to the ends resulting in regions of the transposon arrays lacking silence chromatin. On the other hand, the level of capping proteins might be sufficient to cover a much smaller region still ensuring normal end protection.

**Fig 9:**
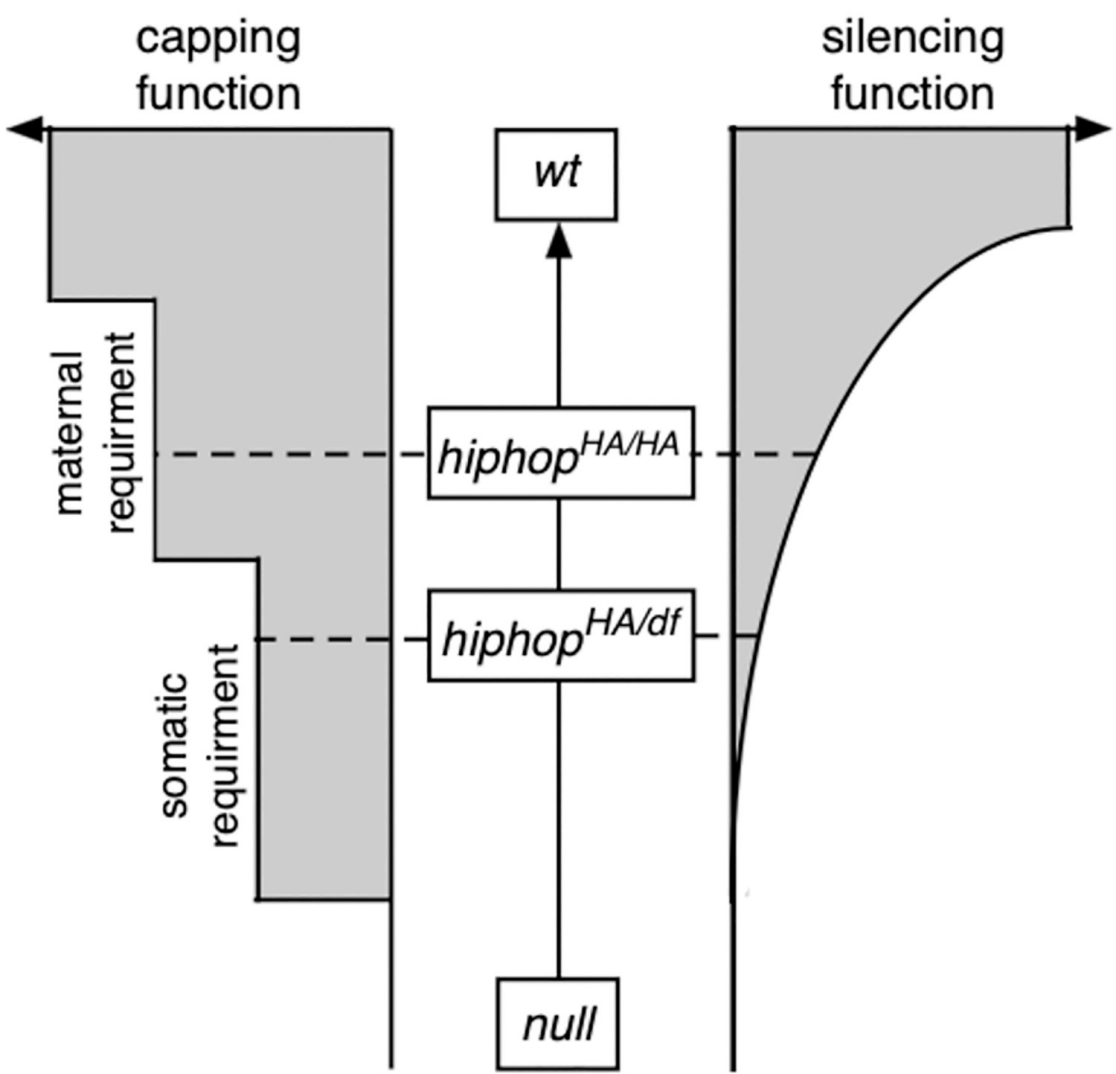
A speculative model for the intricate relationship between the capping and silencing functions of the capping machinery. The scale in the center represents the strengths of various *hiphop* mutant combinations from “*null*” to “*wt*”, with the deduced positions indicated for the *hiphop*^*HA/HA*^ and the *hiphop*^*HA/df*^ allelic combinations. The capping and silencing functions are shown to either side of the center scale as two horizontal scales. For the capping function, two thresholds of functional requirements have been identified: one for capping in early embryos that are controlled by the maternal *hiphop* genotype, and the other for capping during somatic development. For the silencing function, a continuous decline correlates with the strengths of different mutations. It is defined as lost (reaching zero) when viability of the mutants is no longer supported due to somatic uncapping. Depending on which function is more sensitive to the partial loss of HipHop functions, separation-of-function mutations would have different properties. This model assumes that the silencing function has a lower threshold of producing mutant phenotypes than one for capping.

Therefore, it is conceivable that both transposon de-repression and embryonic uncapping are caused by the same partial loss of HipHop function in our mutant. Based on this proposition we raise a model depicted in Fig 9 that is consistent with the empirical data presented in this study. The model assumes that the silencing function is more sensitive to the perturbation of HipHop, and it predicts that no individual mutation would be defective in capping but proficient in transposon silencing. Such a prediction might be tested with a systematic mutagenesis of *hiphop* or *cav*. Since end capping might be considered as a more conserved function of HipHop or HOAP, and since neither the viable *cav* allele reported by Saint-Leandre et al. [24] nor our *hiphop*^*HA*^ allele entails replacement of highly conserved residues in the protein sequences, we predict that separation-of-function alleles likely arise from changes of these more conserved residues, a direction that we are actively pursuing.

### HipHop as a part of the host machinery restricting sites of retro-transposition

There does not appear to be an optimal length of telomeric arrays in *Drosophila* as mounting evidence suggest that *Drosophila melanogaster* can sustain a large variation in copy numbers of these elements. Therefore, “extra-long” telomeric arrays do not necessarily pose a grave fitness cost in flies. On the other hand, a telomeric element would be a significant detriment to host fitness had it gained the ability to insert elsewhere in the genome. Restricting the insertional site, but not the expression level, of these elements might represent the most important tool that the host evolves to balance the potential cost of having active transposons. This proposition is prompted by the remarkable finding that homozygous stocks of *hiphop*^*HA*^ does not accumulate longer telomeric arrays over generations even in the continuing presence of excess amount of transposon RNPs. This contrasts with the outcome in previous examples with a similar condition of telomere hyperactivation (e.g. [21–23]). Our results suggest that the same *hiphop* mutation that leads to telomeric de-repression also suffers a partial loss in its ability to recruit the machinery needed for transposition.

The mechanism for transposon targeting to chromosome ends remains poorly understood for the *Drosophila* elements. Our prior study suggests a multi-faceted mechanism that directs the formation of an elaborate structure of transposon RNP, which is both cell-cycle regulated and coincides with on-going telomeric transcription [5]. More importantly, we provided the first line of evidence that the host capping complex is intimately involved in transposon recruitment to the ends [5,10].

How is it then that the elements unable to land at the telomere in *hiphop*^*HA*^ mutants also do not insert somewhere else in the genome? It has been proposed that *Drosophila* telomeric elements are actively targeted to a gene-poor region of the genome [29,54]. In other similar cases of transposon targeting, host factors are known to be essential for the process as expected. For examples, the Ty elements in budding yeast target RNA pol III transcribed tRNA genes by interacting with subunits of the polymerase complex (reviewed in [55]). The fission yeast Tbf1 elements are targeted to genomic regions specifically bound by the host Sap1 protein [56,57]. Remarkably, loss of Sap1 drastically reduces Tbf1 transposition efficiency suggesting that Sap1 not only targets but is itself necessary for Tbf1 transpositions. We speculate that HipHop plays a similar role for the transposition of telomeric elements in *Drosophila* so that most of the excess transcripts in a *hiphop*^*HA*^ mutant germline are unable to insert anywhere in the genome due to the same partial loss of HipHop function. Our model predicts that the same *hiphop*^*HA*^ mutation should also prevent over-elongation of telomeres under another genetic background that is permissive to over-elongation, e.g., the *Tel* background [21]. Unfortunately, a combination of *hiphop*^*HA*^ and *Tel* causes female sterility complicating the test of the hypothesis.

## Materials and methods

### Drosophila stocks and crosses

Fly stocks and crosses were cultured at room temperature on standard cornmeal food, and *w*^*1118*^ was used as the wide type stock in our study except otherwise noted. The following stocks were obtained from the Bloomington Drosophila Stock Center with the stock numbers shown in parentheses: *spnE*^*1*^ (3327), *spnE*^*hls-Δ125*^ (43638), *aub*^*HN2*^ (8517), *aub*^*QC42*^ (4968), *In(1)w*^*m4h*^ (6234), *Df(3L)Cat* (2990, a deficiency of *hiphop*), *Df(2L)BSC227* (9704, a deficiency of *Su(var)205*), *Df(2R)Exel7149* (7890, a deficiency of *rhino*), *Df(2L)Exel7077* (7850, a deficiency of *chk2*). The *Gaiano (Tel)* stock was a gift from Dr. Jim Mason at NIEHS [21]. The *chk2*^*p30*^ stock was a gift from Dr. Pam Geyer of the University of Iowa [58]. The *hiphop*^*L41*^ and *mre11*^*58S*^ mutations have been described previously [31,41].

The *hiphop*^*HA*^ allele was generated by the SIRT gene targeting method [59]. Briefly, the Rong and Golic [60] gene targeting by homologous recombination scheme was employed to insert an *attP* landing site for the phiC31 site specific integrase 1998 bp upstream of the ATG codon of *hiphop*. A plasmid carrying the *3xHA* tagged *hiphop* locus and an *attB* site was then inserted at the *attP* site creating a duplicated *hiphop* locus. An I-CreI mediated reduction of this tandem duplication resulted in the *hiphop*^*HA*^ allele used in this study. All mutations and coding regions were sequenced.

### Transgenic constructs and fly lines

The *hiphop* rescuing construct contains a 3kb genomic fragment (3L: 18820210–18823346), which was cloned into the pUAST-attB vector and inserted into chromosome *2* (25C6) by phiC31-mediated transformation. A full length *HeT-A* element was subcloned from the BAC clone 44B08 (bacpacresources.org) into pBluescript. A *gfp* fragment was inserted downstream of the *orf1* coding region by recombineering [61] and this *gfp*-tagged *HeT-A* full length element was inserted into pW8 vector for P element mediated transformation. Insertions on chromosome *3* and chromosome *X* were used. Primers used for cloning are listed in S3 Table.

### Fertility, fecundity and hatching rate tests

Female fertility tests were performed as described [22]. Briefly, individual 1–2-day old females were crossed to three *w*^*1118*^ males at room temperature. The parents were transferred after 5 days, and total progenies were counted. Female fecundity tests were performed with ten 1–2-day old females and twenty *w*^*1118*^ males at room temperature. Eggs were collected on grape juice plates every 24h and counted. For hatching rate tests, 1–5-day old females were crossed to *w*^*1118*^ males at 25°C. Eggs were collected on grape juice plates every 2h. Plates were maintained at 25°C for 24–48 hours and hatched animals were counted.

### mRNA-seq experiments, mRNA-seq analysis and qPCR

#### sample preparation for mRNA-seq

Males from *hiphop*^*HA/HA*^, *Df(3L)Cat/TM6B* and *w*^*1118*^ stocks were mated to *w; Sb/TM6B* females separately. F1 males that were *w; hiphop*^*HA*^*/TM6B*, *w; Df(3L)Cat/TM6B* or *w; hiphop*^*+*^/TM6B were mated to *w; Sb/TM6B* females again to recovered F2 males that were of the same genotypes as their F1 fathers. These crosses were repeated for two more times. At last, males were crossed to *hiphop*^*HA/HA*^ females to generate *hiphop*^*HA/HA*^, *hiphop*^*HA/Df*^ and *hiphop*^*HA/+*^ females for ovary harvesting. Ovaries were dissected from females kept at room temperature for 15 days and used in total RNA extraction. Three biological replicates were sent for library construction followed by sequencing performed by Novogene Corporation of China.

#### mRNA-seq analysis

RNA-seq analysis were performed as described [22]. Briefly, adapter sequences were trimmed by Cutadapt. Reads with a Phred quality score less than 20 and with length less than 36bp were removed. The remained reads were first mapped to the consensus-sequence database by Satyaki et al. [22] using Bowtie2, then the filtered-out reads were mapped to unmasked *D*. *melanogaster* genome using Bowtie2 again. We generated the TE count and genomic count metrics with HTSeq and performed differential expression analysis using the DESeq2 package.

#### RT-qPCR and qPCR

RNA was extracted following standard TRIZOL (Ambion, 15596018) RNA isolation protocol. Reverse transcription was performed by PrimeScript RT reagent Kit (TAKARA, RR047A). For DNA extraction, adults or dechorionated embryos were homogenized in lysis buffer (100mM Tris-HCl, 50mM NaCl, 100mM EDTA and 1% SDS) with 30μg/ml of RNase A. Samples were lysed at 65°C for 1h and then subjected to phenol-chloroform extraction.

#### qPCR quantification

Absolute quantification was used to analyze transposon expression levels and their DNA copy numbers. Primers are listed in S3 Table. For making standard curves, qPCR products were first cloned into the pMD18-T vector (TAKARA, 6001) and used as standard qPCR templates. qPCR reactions were carried out on a Real-time PCR machine (ABI, QuantStudio 5) following the protocol provided in the SYBR FAST qPCR Kit (KAPA, KK4601).

### Isolation of telomere fusion junctions

Experiments were performed as described [41]. Briefly, females with the desired genotype were crossed to *w*^*1118*^ male at room temperature and 0–2 h embryos were collected for DNA extraction. Primers used were HeT-A453rev (primer 1), HeT-A1196rev (primer 2), HeT-A1751rev (primer 3) and HeT-A1997rev (primer 4).

### Chromatin immunoprecipitation

ChIP was performed as described [62]. For each IP experiment ~ 60 pairs of ovaries were dissected. A mouse anti-HipHop raised against the full-length recombinant protein and a mouse anti-HP1 antibody (C1A9 from DSHB) were used for ChIP. ChIP results were analyzed as described [6]. Primers are listed in S3 Table.

### Immunostaining, FISH and Western blot

#### Immunostaining and mitotic chromosome squash

Embryo fixation and DAPI staining were performed by standard protocols with a fixative of a 1:1 mixture of 4% formaldehyde in PBS and heptane for 20 minutes. Larval brains were fixed with 3.7% formaldehyde in PBS and stained with a 1:200 dilution of a guinea pig anti-ORF1p [5]. Salivary glands were dissected from third instar larvae in PBS and fixed with 2% formaldehyde in 45% acetic acid for 5 min. Immunostaining of polytene chromosomes was performed with standard protocols using a rabbit anti-HOAP and a mouse anti-HipHop antibodies at 1:200. Mitotic spreads of larval and embryonic nuclei were performed as describe [41].

#### Western blotting

Ovaries were dissected in PBS and then boiled with SDS-PAGE loading buffer. Rabbit anti-HOAP and guinea pig anti-ORF1p were used at 1:1000. Mouse anti-HP1 (DSHB, C1A9) antibody was used at 1:200 and mouse anti-α-tubulin (Sigma, DM1A) at 1:10000.

## Supporting information

S1 Fig Normal capping complex localization in *hiphop* somatic cells. **A**. At the left are RT-qPCR results measuring *hiphop* transcript levels in the ovaries. On the right is a measurement of HP1 and HOAP levels in larval extracts using Western blotting. **B**. Immunostaining of HipHop^HA^ and HOAP on polytene chromosomes in salivary glands from *hiphop*^*HA/df*^ larvae. The boxes outline areas of which magnified images are shown in **C**.
(TIF)

S1 Table *hiphop*^*HA*^ does not cause lethality. (DOCX)

S2 Table Expression analysis of other TEs in *hiphop* mutant germline. (XLSX)

S3 Table Primer list. (DOCX)

S1 Data Raw data used for drafting the graphs in all figures. (XLSX)
