# Supplementary figures and images for: Taming active transposons at Drosophila telomeres: The interconnection between HipHop’s roles in capping and transcriptional silencing
Source: PLoS Genet. 2021 Nov 23;17(11):e1009925. doi: 10.1371/journal.pgen.1009925 (PMC8651111; doi:10.1371/journal.pgen.1009925)

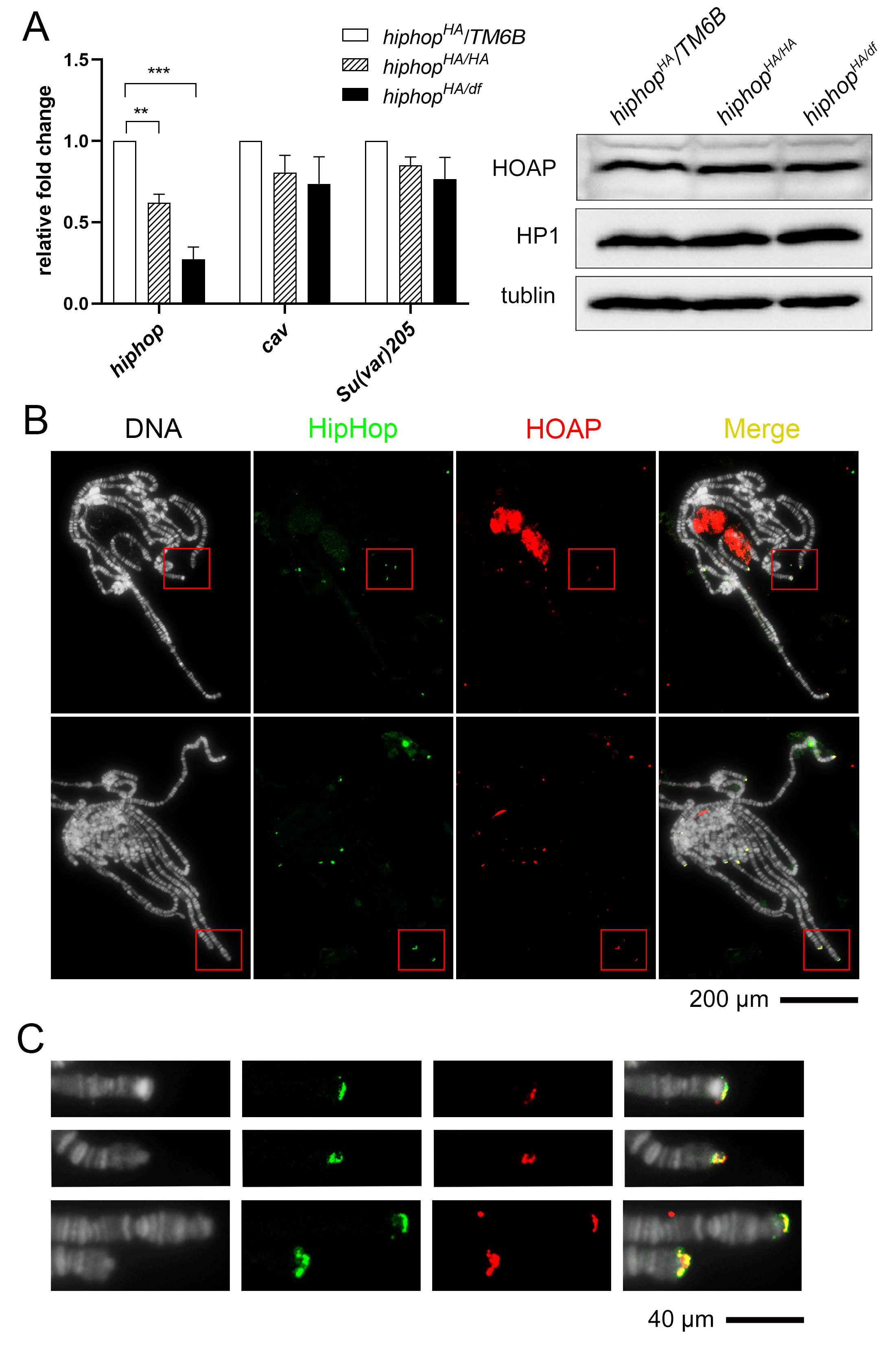

Supplement: S1 Fig — A. At the left are RT-qPCR results measuring hiphop transcript levels in the ovaries. On the right is a measurement of HP1 and HOAP levels in larval extracts using Western blotting. B. Immunostaining of HipHopHA and HOAP on polytene chromosomes in salivary glands from hiphopHA/df larvae. The boxes outline areas of which magnified images are shown in C. (TIF) [file pgen.1009925.s001.tif]
