# Supplementary material for: Taming active transposons at Drosophila telomeres: The interconnection between HipHop’s roles in capping and transcriptional silencing
Source: PLoS Genet. 2021 Nov 23;17(11):e1009925. doi: 10.1371/journal.pgen.1009925 (PMC8651111; doi:10.1371/journal.pgen.1009925)
Supplement: S1 Table — (DOCX) [file pgen.1009925.s002.docx]

S1 Table. *hiphop^HA^* does not cause lethality

| Father | *hiphop^HA^/TM6* | | *Hiphop^df^/TM6* | |
| --- | --- | --- | --- | --- |
| Mother | *hiphop^HA^/TM6* | | *hiphop^HA^/TM6* | |
| Progeny | *hiphop^HA/HA^* | 102 | *hiphop^HA/df^* | 195 |
|  | *hiphop^HA^/TM6* | 260 | *hiphop ^a^/TM6* | 422 |
|  | Ratio^b^ | 0.39 | Ratio^b^ | 0.46 |

^a^: *hiphop^HA^/TM6* and *hiphop^df^/TM6*

^b^: TM6/TM6 did not survive. The expected ratio is 0.50.
