# Supplementary material for: Taming active transposons at Drosophila telomeres: The interconnection between HipHop’s roles in capping and transcriptional silencing
Source: PLoS Genet. 2021 Nov 23;17(11):e1009925. doi: 10.1371/journal.pgen.1009925 (PMC8651111; doi:10.1371/journal.pgen.1009925)
Supplement: S3 Table — (DOCX) [file pgen.1009925.s004.docx]

S3 Table Primer List

| **Primer Name** | **Primer Sequence** |
| --- | --- |
| For *hiphop* rescue (homologous sequence are underlined) | |
| Hiphop-FP40 | TTAACAGATCTGCGGCCGCGGCTCGAGTTCACTTGTCTCGCACACTC |
| Hiphop-RP1118 | CATCTGGAATAGATCATGCACC |
| GFP-FP7 | GGTGCATGATCTATTCCAGATGAGCAAGGGCGAGGAGCTGTT |
| GFP-RP717 | GCGAGCCCTCGTCAATGGAGGCACCTCCACCCTTGTACAGCTCGTCCATGC |
| Hiphop-FP1140 | GCCTCCATTGACGAGGGCTC |
| Hiphop-RP3157 | AAGGTTCCTTCACAAAGATCCTCTAGATAGACTAAGGTAAGAGGTCG |
| For telomere fusion detection | |
| HeT-A453rev | ACTTCCCATTGCATCGCTCGTT |
| HeT-A1196rev | GGCGGAAAAATGCTGGGAGTTAC |
| HeT-A1751rev | CGCATGGGGCCACCTGTAG |
| HeT-A1997rev | GTGGCGGGGGTGGTTCTTG |
| For qRT-PCR and qPCR |  |
| RP49_F | ATGACCATCCGCCCAGCATAC |
| RP49_R | GCTTAGCATATCGATCCGACTGG |
| HeTA_ORF1_F | CCAGGCAAGCGGACAAACGA |
| HeTA_ORF1_R | GGAGTGATGAGCGGCGGAAA |
| HeTA_ORF2_F | GGCCAACGGACTTCCTTTATTCAC |
| HeTA_ORF2_R | CGCATGGGGCCACCTGTAG |
| HeTA_3UTR_F | CGCGCGGAACCCATCTTCAGA |
| HeTA_3UTR_R | CGCCGCAGTCGTTTGGTGAG |
| TART_F | ATCTGTCTACTGTCCGCCTTCGCTA |
| TART_R | AATGAACTTTGTCTGCCCTCCCA |
| TAHRE_F | CATCAGACGAATCATAAACGCC |
| TAHRE_R | GATAAGGAGGTCATATATTAAAGGG |
| I-element_F | GCATCCCTCAACTTCTCCTCCACAG |
| I-element_R | ACAAAATCACTTCAAAAACATACCAATCCC |
